# Gastrointestinal perforation secondary to COVID-19: Case reports and literature review

**DOI:** 10.1097/MD.0000000000025771

**Published:** 2021-05-14

**Authors:** Reem J. Al Argan, Safi G. Alqatari, Abir H. Al Said, Raed M. Alsulaiman, Abdulsalam Noor, Lameyaa A. Al Sheekh, Feda’a H. Al Beladi

**Affiliations:** Department of Internal Medicine-College of Medicine-Imam Abdulrahman Bin Faisal University -King Fahd Hospital of the University-Khobar-Eastern Province-Saudi Arabia.

**Keywords:** acute abdomen, acute diverticulitis, cecal mass, corona virus disease-2019, gastrointestinal perforation

## Abstract

**Introduction:**

Corona virus disease-2019 (COVID-19) presents primarily with respiratory symptoms. However, extra respiratory manifestations are being frequently recognized including gastrointestinal involvement. The most common gastrointestinal symptoms are nausea, vomiting, diarrhoea and abdominal pain. Gastrointestinal perforation in association with COVID-19 is rarely reported in the literature.

**Patient concerns and diagnosis:**

In this series, we are reporting 3 cases with different presentations of gastrointestinal perforation in the setting of COVID-19. Two patients were admitted with critical COVID-19 pneumonia, both required intensive care, intubation and mechanical ventilation. The first one was an elderly gentleman who had difficult weaning from mechanical ventilation and required tracheostomy. During his stay in intensive care unit, he developed Candidemia without clear source. After transfer to the ward, he developed lower gastrointestinal bleeding and found by imaging to have sealed perforated cecal mass with radiological signs of peritonitis. The second one was an obese young gentleman who was found incidentally to have air under diaphragm. Computed tomography showed severe pneumoperitoneum with cecal and gastric wall perforation. The third case was an elderly gentleman who presented with severe COVID-19 pneumonia along with symptoms and signs of acute abdomen who was confirmed by imaging to have sigmoid diverticulitis with perforation and abscess collection.

**Interventions:**

The first 2 cases were treated conservatively. The third one was treated surgically.

**Outcome:**

Our cases had a variable hospital course but fortunately all were discharged in a good clinical condition.

**Conclusion:**

Our aim from this series is to highlight this fatal complication to clinicians in order to enrich our understanding of this pandemic and as a result improve patients’ outcome.

## 1 Introduction

Corona virus disease-2019 (COVID-19) had been declared pandemic in March 2020.^[1]^ It presents most commonly with fever in more than 80% of cases followed by respiratory symptoms which could progress to adult respiratory distress syndrome in critical cases.^[2]^ However, extra respiratory manifestations are being frequently recognized in association with COVID-19.^[3]^ The gastrointestinal (GI) manifestations have been reported in descriptive studies from China.^[2]^ The most frequently reported GI symptoms are nausea, vomiting, diarrhoea, and abdominal pain.^[2,4,5]^ However, it is rarely reported for COVID-19 to present with GI perforation. To the date of writing this report, there have been only 13 reported of GI perforation in association with COVID-19.

In this series, we are reporting 3 cases who developed GI perforation in association with COVID-19. The first 2 cases developed this fatal complication after presenting with critical COVID-19 pneumonia which required intensive care unit (ICU) admission and mechanical ventilation. The third case presented with severe COVID-19 pneumonia and was diagnosed to have GI perforation at the time of presentation. The first 2 cases were managed conservatively, and the third case was managed surgically. All of the 3 cases recovered and were discharged in good condition. We are reporting this series in order to highlight this rare but fatal complication of COVID-19. This will enhance awareness of clinicians to such complication where early diagnosis and management is crucial in order to improve the patients’ outcome.

## 2 Case reports

### 2.1 The patients provided informed consent for publication of their cases

#### 2.1.1 First case

**Figure 1:**
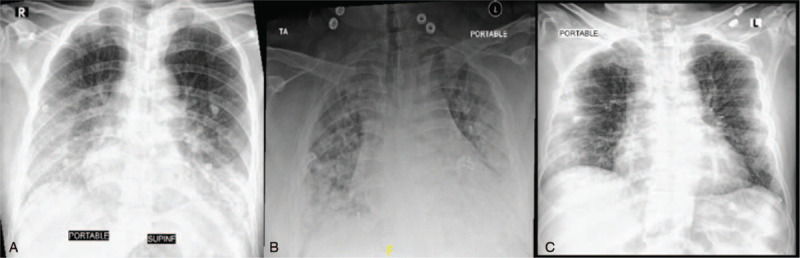
The chest X-ray (CXR) of the 3 cases at the time of presentation. (A): CXR of the 1st case showing bilateral lower lung fields air apace opacities. (B): CXR of the 2nd case showing bilateral scattered air space consolidative patches throughout the lung fields predominantly over peripheral and basal lungs. (C): CXR of the 3rd case showing bilateral middle and lower zones peripheral ground glass opacities.

A 70-year old male patient known to have type 2 diabetes mellitus (T2DM), presented to our emergency department (ED) on 1st of June 2020 complaining of 3-day history of dry cough and fever. On examination: Vital signs were remarkable for tachypnea with respiratory rate (RR): 28/min and desaturation with oxygen saturation (O2 sat):81% on room air (RA) but maintained >94% on 15 litres of oxygen via a non-rebreather mask. Nasopharyngeal swab tested positive for SARS-CoV-2 polymerase chain reaction (PCR). Chest X-ray (CXR) showed bilateral lower lung fields air apace opacities (Fig. 1A) consistent with COVID-19 pneumonia. Laboratory investigations were remarkable for high Lactate dehydrogenase (LDH), inflammatory markers, D-dimer and markedly elevated Ferritin (Table 1). He was started on Methylprednisolone 40 mg IV BID, Hydroxychloroquine, Ceftriaxone, Azithromycin, Oseltamivir, and Enoxaparin. After 5 days of hospital admission, he deteriorated and could not maintain saturation on non-rebreather mask, so he was shifted to ICU, intubated and mechanically ventilated. Ceftriaxone was upgraded to Meropenem in addition to same previous therapy. COVID-19 therapy was stopped after completing 10 days, but he was continued on steroids.

**Table 1:** The laboratory investigations of the 3 cases on presentation.

Test	First case	Second case	Third case	Normal range
Complete Blood Count				
White Blood cells	6.4	4.2	5.7	(4.0–11.0) K/uI
Hemoglobin	15.1	12.1	13.4	(11.6–14.5) g/dL
Platelets	147	232	283	(140–450) K/uI
Renal Profile				
Blood urea nitrogen	10	14	11	(8.4–21) mg/dL
Creatinine	0.92	0.82	0.82	(0.6–1.3) mg/dL
Liver Profile				
Total Bilirubin	0.5	0.5	1.0	(0.2–1.2) mg/dL
Direct Bilirubin	0.3	0.2	0.3	(0.1–0.5) mg/dL
Alanine Transferase (ALT)	26	52	41	(7–55) U/L
Aspartate transferase (AST)	42	50	52	(5–34) U/L
Alkaline phosphatase (ALP)	74	55	74	(40–150) U/L
Gamma-glutamyl transpeptidase (GGTP)	53	21	39	(12–64) U/L
Lactate dehydrogenase (LDH)	434	442	617	(81–234) U/L
Inflammatory Markers				
Erythrocyte Sedimentation rate (ESR)	63	101	49	0–10 mm/h
C-Reactive Protein (CRP)	7.92	18.32	10.78	0–5 mg/dL
Others				
Ferritin	1114.72	565.86	654.87	(21.81–274.66) ng/mL
D-Dimer	0.6	0.41	1.66	<=0.5 ug/mL

Multiple trials of weaning from mechanical ventilation failed. So, tracheostomy was carried out on 20th day of ICU admission and then he was successfully extubated. During his stay in ICU, urine analysis was persistently positive for urinary tract infection secondary to *Candida Abican*. So, he was started on Caspofungin. At that time, blood culture was negative. After 4 days of Caspofungin, urine analysis and culture became negative. On 32nd day of hospital admission, he was stable clinically, requiring 40% FiO2 through tracheostomy mask, so he was transferred to COVID-19 isolation ward. Meropenem was stopped after 20 days of treatment. Steroid was tapered after transfer to the ward till it was discontinued after 28 days of therapy.

After 14 days of treatment with Caspofungin, follow up C-reactive protein was persistently high. Thus, full septic workup was requested and revealed *Candida Albican* bacteremia. At that time, urine analysis and culture were negative, Caspofungin was continued for additional 14 days. However, Candidemia persisted, so he was shifted to Anidulafungin for another 14 days. Patient at that time did not have any GI symptoms or signs. For work up of Candidemia, echocardiogram could not be done due to the hospital policy of isolation rooms. Bed side ophthalmology examination was unremarkable.

**Figure 2:**
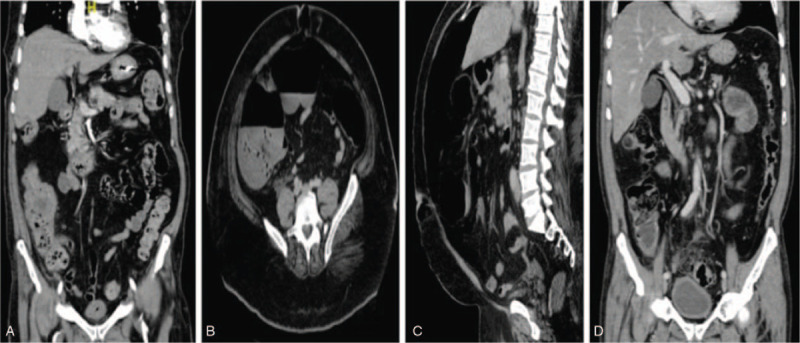
The contrast enhanced computed tomography (CT) of the abdomen of the 3 cases. (A): CT scan abdomen of the 1st case (Coronal image) showing a well-defined rounded heterogeneous enhanced soft tissue mass lesion within the posterior wall of the cecum measuring (3.1 × 3.2 cm) in anteroposterior and transverse diameter associated with discontinuous enhancement of posterior cecum wall and extra-luminal air foci suggestive of complicated perforated sealed cecum mass. This is in addition to adjacent fat stranding with free fluid as well as enhancement of peritoneal reflection suggestive of peritonitis. (B &C): CT scan abdomen of the 2nd case (Axial & Coronal images). (2B): Axial image showing moderate to severe pneumoperitoneum with air seen more tracking along the ascending colon suggestive of a wall defect in the anterior aspect of the cecum. (2C): Coronal image showing a second defect in the stomach wall. (D): CT scan abdomen of the 3rd case (Coronal image) showing severe sigmoid diverticulosis with circumferential bowel wall thickening compatible with acute diverticulitis, small amount of free air compatible with bowel perforation likely arising from the sigmoid colon and a well-defined 3.3 × 1.5 cm abscess collection adjacent to the sigmoid colon.

On 44th day of hospital admission, he developed fresh bleeding per rectum. Hemodynamics were stable. The bleeding resulted in acute drop of 2 g/dL of hemoglobin over 24 hours. He denied abdominal pain, abdominal examination was negative for signs of peritonitis and per rectum examination was unremarkable. Therefore, computed tomography (CT) scan of the abdomen with contrast was carried out. It showed a well-defined mass within the posterior wall of the cecum measuring 3.1 × 3.2 cm associated with discontinuous enhancement and extra-luminal air foci suggestive of complicated perforated sealed cecal mass. This is in addition to radiological findings of peritonitis (Fig. 2A).

In consideration of his stable clinical status, absent signs of peritonitis clinically and being a sealed perforation, he was managed conservatively. So, Meropenem was resumed and Clindamycin was started. 2 days later, bleeding stopped, and he stayed stable clinically without clinical signs of peritonitis. Feeding through nasogastric tube was introduced gradually as tolerated. Antibiotics were continued for a total of 8 days. Trial of weaning from oxygen was attempted gradually which he tolerated till he was maintained on RA. After closure of tracheostomy, on 70th day of hospital admission, the patient was discharged in a good condition with a plan of follow up of cecal mass progression. However, the patient did not follow up in outpatient clinics after discharge.

#### 2.1.2 Second case

A 37-year old male patient, morbidly obese, negative past history, presented to our ED on 11th June 2020. He reported 3-day history of shortness of breath. Vital signs were remarkable for Temperature (Temp.): 38.5 C, pulse rate (PR): 111/min, RR: 30/min and O2 sat: 80% on RA. Laboratory investigations showed high LDH, inflammatory markers and Ferritin (Table 1). He had positive SARS-CoV-2 PCR and CXR showed bilateral air space consolidative patches scattered throughout the lung predominantly over peripheral and basal lungs (Fig. 1B). He was admitted to COVID-19 isolation ward as a case of COVID-19 pneumonia and started on: Triple therapy in the form of: Interferon B1, Lopinavir/Ritonavir and Ribavirin, in addition to Hydroxychloroquine, Ceftriaxone, Azithromycin, Oseltamivir, Dexamethasone 6 mg IV OD and Enoxaparin.

On the 3rd day of admission, his condition deteriorated so, he was shifted to ICU and intubated because of respiratory failure. He was maintained on same treatment for COVID-19. On 2nd day postintubation, clinically he was vitally stable without active clinical GI signs but a routine follow-up CXR showed air under the diaphragm. Therefore, abdomen CT scan with contrast was carried out and showed moderate to severe pneumoperitoneum with air tracking along the ascending colon suggestive of wall defect at the cecum, in addition to another defect noted in the stomach wall (Fig. 2B & 2C). Ceftriaxone was upgraded to Piperacillin-Tazobactam and Caspofungin was added to cover for possibility of peritonitis. Again, the patient was managed conservatively, since he was asymptomatic. He remained vitally stable without signs of peritonitis. Enteral feeding was started gradually 3 days later and on the 10th day of hospital admission, he was extubated and shifted to COVID-19 isolation ward. COVID-19 therapy was continued for 12 days.

He tolerated feeding very well. Gradual weaning of oxygen supplementation was carried out till it was discontinued. After 14 days of antibiotics, a follow up CT scan of abdomen showed interval resolution of previously seen pneumoperitoneum. He was discharged on 30th day of hospitalization in a good condition.

#### 2.1.3 Third case

A 74-year old male patient known case of T2DM presented to our ED on 17th July 2020. He gave 3-day history of dry cough, shortness of breath and generalized colicky abdominal pain. No other pulmonary or GI symptoms. He had negative past surgical history. Vital signs were remarkable for Temp: 38.4 C, PR: 105/min, RR: 22/min and O2 sat: 90% on RA, required 3 L/min O2 through nasal cannula. Chest examination was remarkable for reduced breath sound intensity bilaterally without added sounds. Abdomen was distended with generalized tenderness and guarding. Blood tests were remarkable for high LDH, inflammatory markers, Ferritin and D-dimer (Table 1). PCR for SARS-COV-2 was positive and CXR showed bilateral peripheral ground glass opacities at middle and lower lung lobes (Fig. 1C). Due to the presence of abdominal pain along with signs of acute abdomen on examination, a CT scan of the abdomen was done. It showed severe sigmoid diverticulosis with radiological findings of acute diverticulitis, free air compatible with bowel perforation likely at the sigmoid colon with 3.3 cm adjacent abscess collection (Fig. 2D).

Therefore, the patient was started on Piperacillin-Tazobactam, Metronidazole in addition to COVID-19 therapy. He underwent emergency exploratory laparotomy. Intra-operatively, pus and fecal peritonitis along with perforation of 0.5 cm at the distal sigmoid colon were found. So, a Hartmann's procedure was performed. Pathology result of resected sigmoid colon revealed diverticular disease with surrounding fibrosis, moderate mucosal inflammation with mixed acute and chronic inflammatory cells, negative for malignancy.

He had smooth postoperative course. Enteral feeding was started on 3rd day postoperation and he improved clinically. After a total of 10 days of hospitalization, supplemental oxygen and antibiotics were discontinued. He was discharged on 11th day of hospitalization in a good condition.

## 3 Discussion

The GI manifestations are the most frequently reported extra-pulmonary manifestations of COVID-19^[2]^ with a prevalence of 10% to 50%.^[4,5]^ The most commonly reported GI symptoms are nausea, vomiting, diarrhoea and abdominal pain.^[2,4,5]^ However, there have been case reports of COVID-19 cases presenting with other GI manifestations. Those include acute surgical abdomen,^[6]^ lower GI bleeding^[7]^ and nonbiliary pancreatitis.^[8]^ In fact, the GI manifestations could be the presenting symptoms of COVID-19 as was reported in a case report by Siegel et al where the patient presented with abdominal pain and upon abdominal imaging, the patient was found to have pulmonary manifestations of COVID-19 in the CT scan of the lung bases.^[9]^

GI perforation is a surgical emergency, carries a significant mortality rate that could reach up to 90% due to peritonitis especially if complicated by multiple organ failure.^[10]^ It can be caused by many reasons. Those include foreign body perforation, extrinsic bowel obstruction like in cases of GI tumors, intrinsic bowel obstruction like in cases of diverticulitis/appendicitis, loss of GI wall integrity such as peptic ulcer and inflammatory bowel disease in addition to GI ischemia and infections.^[11]^ Several infections have been reported to result in GI perforation like *Clostridium difficile, Mycobacterium tuberculosis*, Cytomegalovirus and others.^[12–14]^ COVID-19 have been rarely reported to result in GI perforation. Till the date of writing this report only 13 cases^[15–23]^ have been reported in the literature (Table 2). In addition, Meini et al reported a case of pneumatosis intestinalis in association with COVID-19 but without perforation.^[25]^

**Table 2:** Summary of the previously published cases of gastrointestinal perforation in association with COVID-19.

	First Author [Reference]	Age/ Sex	Co-morbid Conditions	Presenting symptoms	Severity of COVID-19 pneumonia^∗^	COVID-19 Therapy	Symptoms prompted investigations for GI perforation	Site of Perforation	Timing of Perforation post admission	Management of Perforation	Outcome
1	Gonzalvez Guardiola et al ^[15]^	66 Y^†^/ M^‡^	Metabolic syndrome	Not mentioned	Critical	MethylprednisoloneTocilizumab Hydroxychloroquine AzithromycinLopinavir/Ritonavir	Abdominal painIncreased WBC and CRP.	Cecum	Not mentioned	Right colectomy	Not mentioned
2	De Nardi et al ^[16]^	53 Y/M	Hypertension Supra-ventricular tachycardia	FeverCoughDyspnea	Critical	Anakinra Lopinavir/Ritonavir Hydroxychloroquine + Antibiotics	Abdominal pain Abdominal distentionSigns of Peritonitis	Cecum	11^th^ day of admission	Right colectomy & ileo-transverse anastomosis	Discharged Home
3	Kangas-Dick et al ^[17]^	74 Y/M	Negative	FeverDyspneaDry cough	Critical	Hydroxychloroquine +Antibiotics	Increased Oxygen requirementMarkedly distended abdomen	Not specified (CT scan: Not done)	5^th^ day of admission	Conservative	Died
4	Galvez et al ^[18]^	59 Y/M	Status post laparoscopic Roux-en-Y gastric bypass surgery	FeverDry coughMyalgiaHeadacheDyspnea	Moderate	Methylprednisolone + COVID-19 protocol (Not specified)	Acute abdominal painWorsening dyspnea	Gastro-jejunal anastomosis	5^th^ day of admission	Laparoscopy& Graham Patch Repair	Discharged Home
5	Poggiali et al ^[19]^	54 Y/ F^§^	Hypertension	FeverDry coughGERD symptoms	Severe	COVID-19 therapy (Not specified) +Antibiotics	Acute chest pain Painful abdomen	Diaphragm Stomach	At presentation	Surgical Repair	Not mentioned
6	Corrêa Neto et al ^[20]^	80 Y/F	HypertensionCoronary artery disease	Dry coughFeverDyspnea	Critical	COVID-19 therapy(Not specified) +Antibiotics	Diffuse abdominal pain & stiffness	Sigmoid	At Presentation	Laparotomy with recto-sigmoidectomy & terminal colostomy	Died
7	Rojo et al ^[21]^	54 Y/F	HypertensionObesityDyslipidemiaEpilepsy	Cough,MyalgiaCostal pain	Critical	Hydroxychloroquine Lopinavir/Ritonavir MethylprednisoloneTocilizumab	FeverHemodynamic instabilityAnemia	Cecum	15^th^ day of admission	Laparotomy with right colectomy and ileostomy	Died
8	Kühn et al ^[22]^	59 Y/M	Not mentioned	FeverNauseaAbdominal pain Fatigue, Headache	Not specified	Not mentioned	Abdominal pain	Jejunal diverticulum	At presentation	Open small bowel segmental resection & anastomosis	Discharged Home
9	Seeliger et al ^[23]^	31Y/M	Not mentioned	Dyspnea	Severe	Not mentioned	Not mentioned	Left colon	At presentation	Left Hemicolectomy	Discharged Home
10		82 Y/F		Dyspnea, Diarrhoea	Critical			Sigmoid	At presentation	Open drainage of peritonitis	Died
11		71 Y/F		Fever	Severe			Gangrenous appendix	At presentation	Laparoscopic appendectomy	Discharged Home
12		80Y/M		Not mentioned	Severe			Sigmoiditis	At presentation	Hartmann procedure	Discharged Home
13		77 Y/M		Dyspnea	Critical			Duodenal ulcer	23rd day of admission	Open duodenal exclusion, omega gastro-enteric anastomosis	Died
14	This Report	70Y/M	T2DM	FeverCough	Critical	Methylprednisolone HydroxychloroquineOseltamivir Enoxaparin+Antibiotics	Bleeding per rectumHemoglobin Drop	Cecal mass	44th day of admission	Conservative	Discharged Home
15		37Y/M	Morbid obesity	Dyspnea	Critical	Interferon B1Lopinavir/RitonavirRibavirinHydroxychloroquineOseltamivirDexamethasone+Antibiotics	Air under diaphragm was found incidentally in a follow up CXR	Cecum	4th day of admission	Conservative	Discharged Home
16		74Y/M	T2DM	CoughDyspnea Abdominal pain.	Severe	Lopinavir/RitonavirRibavirinMethylprednisolone+Antibiotics	Abdominal painSigns of peritonitis	Sigmoid diverticulosis/diverticulitis	At presentation	Exploratory laparotomy with Hartmann's procedure	Discharged Home

∗ Severity of COVID-19 pneumonia is based on classification of severity by Ministry of Health-Saudi Arabia.^[24]^

† Y = Year.

‡ M = Male.

§ F = Female.

Most of the previously reported cases presented initially with respiratory symptoms, 4 cases had also GI symptoms at presentation in the form of abdominal pain, stiffness, nausea and diarrhoea^[19,20,22,23]^ [Table 2]. Eleven out of the 13 cases had severe-critical pneumonia that required either high flow oxygen, intubation or mechanical ventilation which is similar to our first 2 cases. This may indicate that GI perforation is more common in severe and critically ill COVID-19 cases. The most common symptoms which prompted investigations for bowel perforation were abdominal pain and distention [Table 2]. Other indications were signs of peritonitis,^[16]^ worsening hemodynamics^[17,18,21]^ and rising inflammatory markers.^[15]^

Only one of our cases had abdominal pain and tenderness at presentation. Another developed anemia due to active lower GI bleeding which is similar to the case published by Rojo et al^[21]^ where the patient developed anemia and found to have hemoperitoneum with pericecal hematoma. This is probably explained by the site of perforation since both had cecal perforation. Our other case was diagnosed incidentally after demonstration of air under diaphragm in routine CXR. GI perforation was diagnosed from first day up to 23rd day of presentation with COVID-19 [Table 2]. Our patients had similar variable timing of GI perforation in relation to presentation with COVID-19. It ranged from the first day of diagnosis up to 40 days after presentation with COVID-19 pneumonia. This may tell us that GI perforation could happen at any time during the course of the infection. Our report demonstrates different presentation of GI perforation with COVID-19 since in 2 of the 3 cases, the infection predisposed to having perforation of an underlying GI lesions (cecal mass and diverticulosis). Only Kuhn et al reported similar presentation where the patient had perforation of jejunal diverticulum.^[22]^ This may tell us that having COVID-19 predispose patients with underlying GI lesions to perforation. In addition, in our first case, we think that the source of Candidemia was most probably the bowel since it was persistent even after clearance of *Candida Albican* from the urine, but it was overlooked due to the absence of GI symptoms at the time of developing the Candidemia. In a study of 62 cases with peritonitis secondary to gastric perforation, Candida species was isolated in 23 cases in peritoneal fluid culture.^[26]^ Therefore, in presence of Candidemia especially in absence of clear source, evaluation of the bowel as a potential source should always be kept in mind.

The effect of SARS-COV-2 virus on the GI system can be explained by different mechanisms. First, the virus uses the same access to enter respiratory and GI tract epithelium which are Angiotensin converting enzyme 2 receptors giving the virus the chance to replicate inside GI cells.^[27]^ In addition, faecal-oral transmission has also been postulated, due to the presence of viral RNA in stool samples.^[28]^ Perforation could result from altered colonic motility due to neuronal damage by the virus^[29]^ in addition to local ischemia resulting from hypercoagulable state caused by the virus especially in critically ill patients.^[30]^ Corrêa Neto et al reported finding ischemia of the entire GI tract during exploratory laparotomy for sigmoid perforation with COVID-19.^[20]^ In addition, Rojo et al reported presence of microthrombi and wall necrosis in the pathology examination of his COVID-19 case with bowel perforation.^[21]^ Other possible implicating factors are the use of Tocilizumab and high dose steroids.^[21,31]^ Both are indicated in severe and critically ill COVID-19 cases. Steroids were used in all of our 3 cases since it is indicated in severe COVID-19 pneumonia according Saudi Arabian Ministry of health guidelines^[24]^ but none of our patients received Tocilizumab. Some of these mechanisms could explain the higher risk of GI perforation in severe and critically ill COVID-19 patients.

The diagnosis of GI perforation is based mainly on radiological findings on CT scan. The most specific findings are segmental bowel wall thickening, focal bowel wall defect, or bubbles of extraluminal gas concentrated in close proximity to the bowel wall.^[32]^ Treatment of GI perforation is mainly surgical in order to improve survival.^[33]^ This is in line with the previously published cases where all were managed surgically except the one reported by Kangas-Dick et al due to the patient's critical condition, so he was managed conservatively but unfortunately, he died.^[17]^ However, in selected cases where there are no active signs of peritonitis, abdominal sepsis or having sealed perforation, conservative treatment is an acceptable management strategy.^[34,35]^ This was the case in 2 of our cases who were managed conservatively. Fortunately, they did very well and had good outcome.

## 4 Conclusion

GI manifestations are common in patients with COVID-19. However, GI perforation is rarely reported in the literature. Severe and critically ill COVID-19 patients seem to be at a higher risk of this complication. It has a variable presentation in patients with COVID-19 ranging from incidental finding discovered only radiographically to acute abdomen. The presence of underlying GI lesion predisposes patients with COVID-19 to perforation. High index of suspicion is required in order to manage those patients further and thus, improve their outcome.

## Author contributions

**Conceptualization:** Reem J. Al Argan, Safi G. Alqatari

**Data curation:** Reem J. Al Argan, Abdulsalam Noor, Lameyaa A. Al Sheekh

**Writing – original draft:** Reem J. Al Argan, Lameyaa A. Al Sheekh, Feda’a H. Al Beladi

**Writing – review & editing:** Reem J. Al Argan, Safi G. Alqatari, Abir H. Al Said, Raed M. Alsulaiman
